# RTKN2 Induces NF-KappaB Dependent Resistance to Intrinsic Apoptosis in HEK Cells and Regulates BCL-2 Genes in Human CD4^+^ Lymphocytes

**DOI:** 10.4137/jcd.s2891

**Published:** 2009-09-07

**Authors:** Fiona M Collier, Andrea Loving, Adele J Baker, Janet McLeod, Ken Walder, Mark A Kirkland

**Affiliations:** 1 Barwon Biomedical Research, Geelong Hospital, Barwon Health, Ryrie St, Geelong, Victoria, 3227, Australia.; 2 Department of Malignant Haematology and Stem Cell Transplantation, The Alfred Hospital, Melbourne, Victoria, 3181, Australia.; 3 School of Medicine, Deakin University, Waurn Ponds, Victoria, 3217, Australia.; 4 Metabolic Research Unit, School of Medicine and Institute for Technology Research and Innovation, Deakin University, Waurn Ponds, Victoria, 3217, Australia.

**Keywords:** T-cells, resistance to apoptosis, signaling, NF-KappaB, Bcl-2 genes

## Abstract

The gene for Rhotekin 2 (RTKN2) was originally identified in a promyelocytic cell line resistant to oxysterol-induced apoptosis. It is differentially expressed in freshly isolated CD4^+^ T-cells compared with other hematopoietic cells and is down-regulated following activation of the T-cell receptor. However, very little is known about the function of RTKN2 other than its homology to Rho-GTPase effector, rhotekin, and the possibility that they may have similar roles. Here we show that stable expression of RTKN2 in HEK cells enhanced survival in response to intrinsic apoptotic agents; 25-hydroxy cholesterol and camptothecin, but not the extrinsic agent, TNFα. Inhibitors of NF-KappaB, but not MAPK, reversed the resistance and mitochondrial pro-apoptotic genes, Bax and Bim, were down regulated. In these cells, there was no evidence of RTKN2 binding to the GTPases, RhoA or Rac2. Consistent with the role of RTKN2 in HEK over-expressing cells, suppression of RTKN2 in primary human CD4^+^ T-cells reduced viability and increased sensitivity to 25-OHC. The expression of the pro-apoptotic genes, Bax and Bim were increased while BCL-2 was decreased. In both cell models RTKN2 played a role in the process of intrinsic apoptosis and this was dependent on either NF-KappaB signaling or expression of downstream BCL-2 genes. As RTKN2 is a highly expressed in CD4^+^ T-cells it may play a role as a key signaling switch for regulation of genes involved in T-cell survival.

## Background

Rhotekin 2 (RTKN2) is the recently identified member of the rhotekin proteins.^1^ The two proteins, rhotekin and RTKN2, have homologues in most mammals including human, chimpanzee, horse, mouse, dog and rat; and each of the proteins has an N-terminal Rho-GTPase binding domain (designated HR-1) and a mid-sequence pleckstrin homology (PH) domain.^1^ Although the amino acids are only 65% homologous, the similar protein architecture indicates that they probably share functional characteristics. Rhotekin was discovered in 1996 in an experiment that identified potential RhoA binding proteins.^2^ Since then a number of interacting proteins for rhotekin have been described.^2^–^8^ In particular the murine rhotekin HR-1 domain has been shown to bind to Rho-GTPase (but not to Rac1 or Cdc42) and to inhibit GTPase-activating protein (GAP) activity.^2^ A number of studies have found that rhotekin is involved in functional PDZ protein complexes^9^ dependent on the C-terminal sequence QSPV-COOH^8^,^9^ present in both rhotekin and RTKN2. The interactions between rhotekin and PDZ proteins have suggested roles in gene expression,^8^ neuronal functions,^4^ and cell polarity development.^5^

A specific anti-apoptotic role for rhotekin was reported by a Taiwanese group in two 2004 publications.^10^,^11^ Having identified rhotekin in a majority of gastric cancers tested, and linking it to metastatic progression,^11^ they established a stable rhotekin expressing gastric cell line and showed that the cells were able to withstand apoptosis from sodium butyrate and serum deprivation. Investigation of the anti-apoptotic signaling pathways indicated that NF-kappaB inhibitors (but not PI3-kinase or MAP kinase inhibitors) abrogated the effect.^10^ Also rhotekin over expression lead to induction of a number of NF-kappaB regulated anti-apoptotic genes, cIAP-2, BCL-xL, A1, and A20. Conversely, reducing rhotekin expression by siRNA greatly sensitized cells to apoptosis.^10^ It was concluded that human rhotekin induced cell survival and Rho mediated signaling with the activation of downstream antiapoptotic genes, and may be linked to gastric tumorigenesis.^10^,^11^

Similarly, RTKN2 was identified in cells induced to survive the apoptotic effects of an oxysterol, 25-OHC.^12^ The oxysterols are oxygenated derivatives of cholesterol and have been directly associated with apoptosis in many cell types including hematopoietic and leukemic cells.^13^–^15^ However the link of RTKN2 expression to cell survival was by association only and information on this gene is currently limited to identification of a number of transcribed isoforms and the significant expression in lymphocytic tissues and cells.^1^ RTKN2 is highly expressed in organs comprising sites of lymphopoiesis; the thymus, spleen, bone marrow, lung and colon. In hematopoietic subsets from all sources, peripheral blood (PB), bone marrow (BM) and umbilical cord blood (UCB), expression was restricted to the T-lymphocytes and the immature B-cells derived from the bone marrow.^1^ Further separation of T-cells showed that RTKN2 was differentially expressed in the CD4^+^ T-cells compared to the CD8^+^ cells. Activation of the T-cell receptor (TCR) in CD4^+^ helper T-cells using phytohemagglutinin (PHA) or anti-CD3 induced marked and sustained down regulation of RTKN2 mRNA.^1^ In mice thymic subsets, RTKN2 was approximately 10 to 14-fold higher in immature double negative (CD4^−^/CD8^−^) and double positive (CD4^+^/CD8^+^) immature populations than the single positive mature T-cells (unpublished data).

The gene expression suggests that RTKN2 is a signaling protein that may play a key role in lymphocyte development and survival. The aim of this study was to develop a stable model of RTKN2 over expression in human embryonic kidney (HEK) cells (with a low endogenous expression of RTKN2) and investigate, 1) its role in responses to apoptotic agents and, 2) potential protein interactions; and then use gene knockdown (shRNA) in primary human CD4^+^ T-cells (with high expression levels of RTKN2), to assess viability and responses to apoptosis, and regulated gene expression.

## Results

### Stable expression of RTKN2 in HEK Flp-In cells

#### Confirmation of protein expression

**Figure 1:**
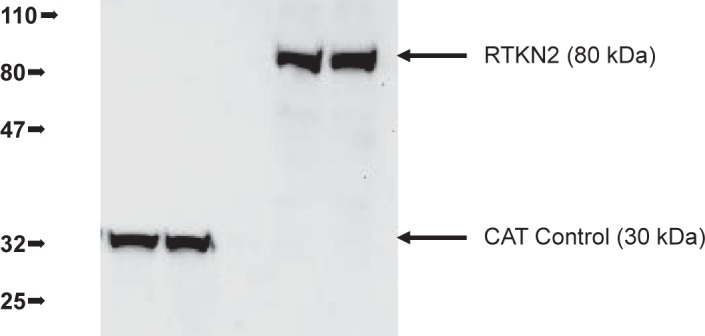
Western blot analysis of stable cell lines to assess expression of RTKN2 and CAT in HEK cells. Three days following the passage of the cells, each of the stable cell lines (CAT#1 and #2, RTKN2#1 and #2) was lysed for protein analysis. 20 ug of total protein from each sample was denatured and run under reducing conditions on a 4%–20% gradient SDS PAGE gel, before transfer and blotting with anti-V5, and anti-mouse HRP. The HRP was visualized using ECL reagent and imaged using the UVP ChemiDoc-It Imaging System (DKSH). The correct sized proteins (CAT, 32 kDa and RTKN2, 80 kDa) are evident in the appropriate lanes.

Stable HEK cells were established with constructs containing either the gene of interest, pcDNA5/FRT-His-V5-RTKN2, or a control gene, pcDNA5/FRT/V5-His/CAT. The control construct expressed the hygromycin resistance gene and chloramphenicol acetyltransferase, (CAT). Three days following the passage of cells, HEK Flp-In stable cells (two controls, CAT #1 and #2, and two genes of interest, RTKN2 #1 and #2) were lysed and analysed by western blot. Each of the over-expressing proteins was tagged with a V5 epitope and clearly visualized using an anti-V5 antibody (Fig. 1). The HEK over expressing cell lines were designated CAT control cells or RTKN2 cells.

**Figure 2:**
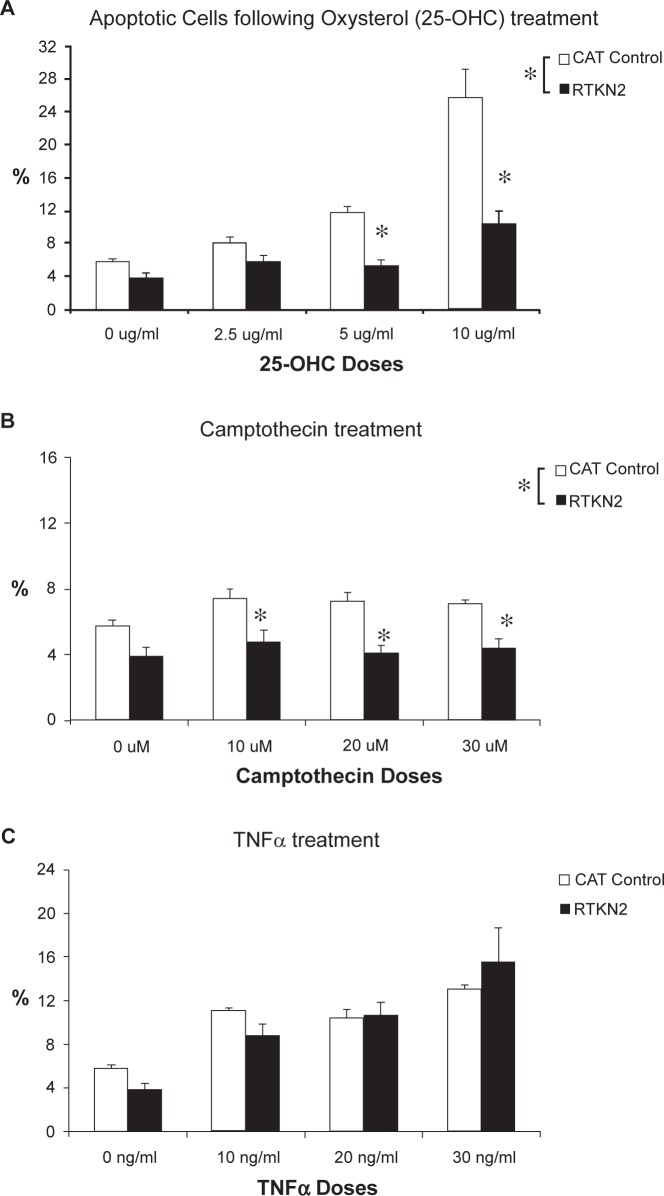
Percentage of apoptotic HEK cells following induction of apoptosis by various agents. Results are expressed as mean ±SEM (n = 6, triplicate treatments/cell line, duplicate cell lines/group). The HEK stable cells were cultured for 48 h in the presence or absence of three apoptotic agents: intrinsic agents, 25-OHC and camptothecin, and the extrinsic agent, TNFα. An aliquot of cells were removed and stained with annexin V and PI to assess the degree of apoptosis. The apoptotic and dying cells were calculated by combining both the percentage of annexin V+ve and PI+ve cells in a gated population. **A**) The RTKN2 cells were overall significantly different to control cells (p < 0.0001, General Linear Model (GLM) in response to 25OH-C. At concentrations of 5 ug/ml and 10 μg/ml they were significantly more resistant than control cells (*p < 0.0001, GLM). **B**) The treatment of Camptothecin failed to induce a dose response however, the results indicated that RTKN2 cells were significantly more resistant at 10 μM (*p = 0.028, GLM), 20 μM (*p = 0.006, GLM) and 30 μM (*p = 0.023, GLM). **C**) The RTKN2 expressing cells were equally sensitive to the apoptotic effects of TNFα.

Response of stable cells to apoptosis induced by oxysterol, camptothecin and TNFαTo determine whether over expression of RTKN2 played a direct role in resistance to apoptosis, three apoptotic agents were chosen and the percentage of cells undergoing apoptotic cell death was measured by flow cytometry using Annexin-V/PI. The oxysterol, 25-OHC, was chosen as resistance to this oxysterol was previously related with elevated levels of RTKN2^1^,^12^ and has been shown to act via the intrinsic pathway.^16^ Camptothecin is a topo-isomerase inhibitor and acts intrinsically via DNA, while TNFα induces extrinsic apoptosis directly via the TNF death receptor. The CAT control and RTKN2 cells were equally sensitive to the extrinsic apoptotic effects of TNFα at all doses (Fig. 2C), while RTKN2 cells were significantly resistant to both the intrinsic agents, oxysterol (25-OHC) (P < 0.0001) (Fig. 2A) and camptothecin (p < 0.0001, main effect between cell lines) (Fig. 2B). Treatment with camptothecin failed to induce a dose response, however the results indicated that RTKN2 cells were significantly more resistant to apoptosis compared to CAT control cells at all doses, 10 μM (p = 0.028), 20 μM (p = 0.006) and 30 μM (p = 0.023) (Fig. 2B).

#### Pathway inhibitor treatments

**Figure 3:**
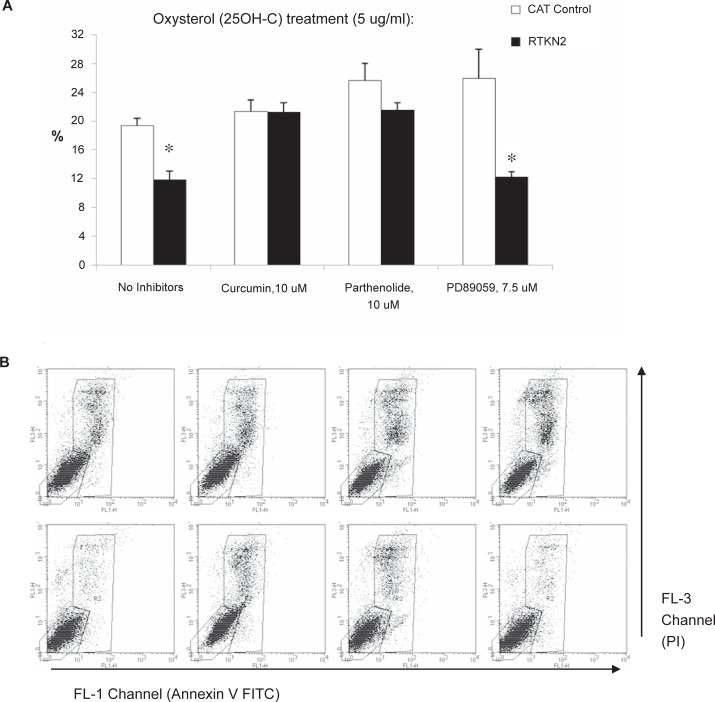
Percentage of apoptotic HEK cells following induction of apoptosis by 25-OHC in combination with various pathway inhibitors. Results are expressed as mean ±SEM (n = 4, duplicate samples/cell line, duplicate cell lines/group). **A**) The HEK stable cells were cultured for 48 h in the presence of the intrinsic apoptotic agent, 25-OHC, and in combination with either curcumin (10 uM), parthenolide (10 uM) (both NF-KappaB inhibitors) or PD98059 (7.5 uM) (MAPK inhibitor). At 48 h an aliquot of cells was removed and stained with annexin V and PI to assess the degree of apoptosis. The apoptotic cells were calculated by combining the percentage of annexin V+ve and PI+ve cells in a gated population. There were significantly less RTKN2 cells undergoing apoptosis after treatment of 25-OHC alone or in combination with PD98059, the MAPK inhibitor (*p = 0.014); but in the presence of both curcumin and Parthenolide, the RTKN2 cells were as sensitive to cell death as the CAT control cells. **B**) The representative flow cytometry dot plots for each of the treatment groups in the bar graphs above. The horizontal axes correspond to the FL-1 green fluorescence channel that detected annexin V FITC, and the vertical axes correspond to the FL-3 red fluorescence channel that detected taining with propidium iodide (PI). The top row of dot plots represents the results for the CAT control cells and the bottom row represents the dot plots for the RTKN2 cells. Region 3 (R3) represents the viable cells (annexin V–ve and PI–ve) and region 2 (R2) represents the apoptotic dying cells (annexin V+ve and PI+ve).

To determine if the apoptotic resistance in RTKN2 cells was mediated via activation of either NF-KappaB or MAPK pathways, the compounds, curcumin and parthenolide (both NF-KappaB inhibitors) and PD89059 (a MAPK inhibitor) were tested in combination with a single dose of the oxysterol, 25-OHC (5 ug/ml). As demonstrated in the previous experiment, there were significantly less RTKN2 cells undergoing apoptosis in the presence of 5ug/ml 25-OHC than the CAT control cells (Fig. 3A and B, no inhibitors, p = 0.014), although due to experimental variability the overall degree of apoptosis was slightly higher than the previous experiment. When treated in combination with NF-KappaB pathway inhibitors, curcumin and parthenolide, the resistance was reversed with RTKN2 cells becoming equally sensitive as the CAT control cells. In contrast PD89059, the MAPK inhibitor, did not alter the level of resistance of the RTKN2 cells (Fig. 3A and B). This was not a generalized effect of the curcumin and parthenolide as there would have been a comparable increase in apoptosis in the CAT control cells.

**Figure 4:**
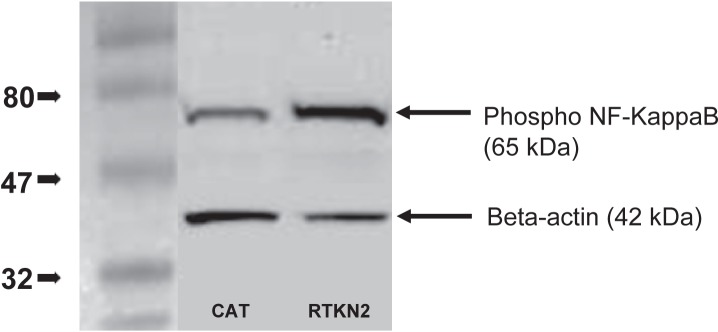
Western blot analysis of phosphorylated NF-KappaB. Three days following passage of the cells CAT control and RTKN2 cells were lysed for protein analysis. The total protein was estimated and approximately 20 ug of each sample was denatured and run under reducing conditions on a 4%–20% gradient SDS PAGE gel, before transfer and blotting with anti-phospho NF-KappaB, and anti-rabbit HRP, The protein loading was quantitated using anti-beta actin. The HRP was visualized using ECL reagent and imaged using the UVP ChemiDoc-It Imaging System (DKSH). The level of phospho-NF-KappaB was greater in the RTKN2 cells.

Increased NF-kappaB activation in RTKN2 cells The activation of NF-KappaB was measured in cultured stable cells three days post-passage. Both control and RTKN2 cells had a visible steady-state level of phospho NF-KappaB but the degree of phosphorylation was greater in the RTKN2 cells (Fig. 4), indicating increased NF-KappaB activation.

#### Expression of apoptotic genes

**Figure 5:**
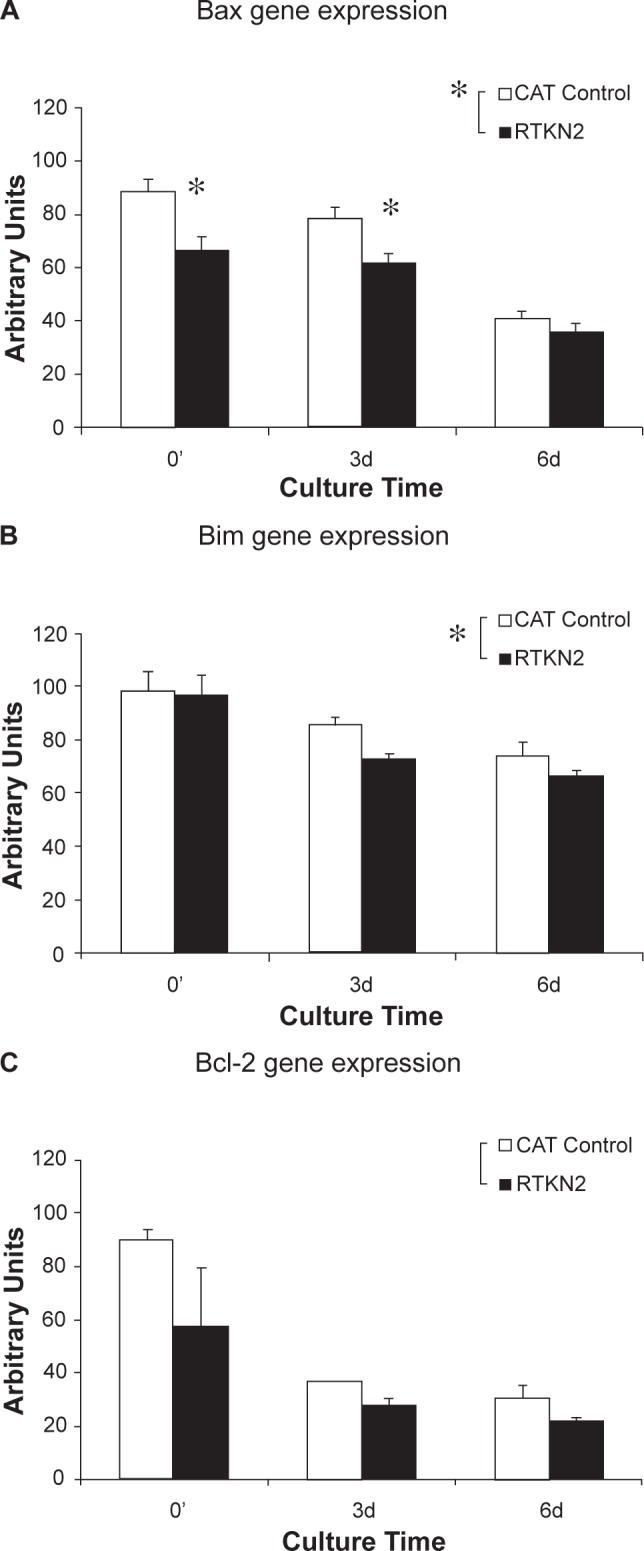
Gene analysis of the HEK stable cells. Results are expressed as mean ±SEM (n = 4). Cells were seeded at equal density and cultured for 6 days with samples taken for RNA extraction at 0, 3 and 6 days. Real time PCR was performed with Applied Biosystems Assays on Demand™ for Bax, Bim and BCL-2, and compared to the mean of two housekeeping genes (β-actin and GAPDH). **A**) Bax gene expression was significantly lower in RTKN2 stables at day 0 (*p = 0.01) and day 3 (*p = 0.03) with a main effect between cell lines (p < 0.0001, GLM). **B**) Expression of Bim was overall significantly lower in RTKN2 cells (p < 0.013, main effect between cell lines, GLM). **C**) There was no significant difference in gene expression of BCL-2 between cell lines.

As the RTKN2 cells were resistant to the two intrinsic apoptotic stimuli (that act directly via the mitochondrial death pathway), the expression of members of the BCL-2 family of genes was assessed by real time PCR in the stable HEK cell lines that had been cultured over a period of 6 days. The expression of the pro-apoptotic gene, Bax, was significantly lower in RTKN2 stables at day 0 (p = 0.01) and day 3 (p = 0.03) with an overall decrease of expression in RTKN2 cells (p < 0.0001, main effect) (Fig. 5A). The expression of Bim (pro-apoptotic) was also overall lower in RTKN2 cells (p < 0.013, main effect) compared to the CAT control cells (Fig. 5B). The expression of BCL-2 was not significantly different between the CAT control cells and RTKN2 cells but did decrease over the 6 day culture period in both cell lines (p = 0.01) (Fig. 5C).

#### Immunoprecipitation with RhoA and Rac2

**Figure 6:**
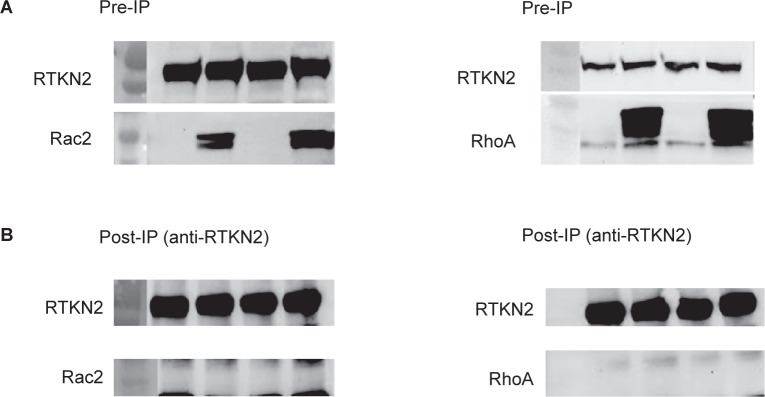
Western blot analysis of HEK RTKN2 stable cell lysates pre- and post-immunoprecipitation (IP) with RhoA and Rac2. A) Pre-IP following transfection with either Rac2 or RhoA, protein lysates were collected (48 h post-transfection) before the immunoprecipitation step (pre-IP) (Fig. 6A). The samples were denatured and run in reducing conditions on a 4%–20% gradient SDS PAGE gel, before transfer and immunoblotting with, anti-V5 (to detect RTKN2), anti-HA (Rac2) or anti-RhoA (RhoA). Bands were detected for all the proteins. B) Post-IP proteins lysates were then collected (48 h post-transfection) and immunoprecipitation performed overnight with anti-V5 (for RTKN2) and A/G sepharose. The samples (Post-IP) were denatured and run in reducing conditions on a 4%–20% gradient SDS PAGE gel, before transfer and immunoblotting with anti-V5 (to confirm IP), anti-HA or anti-RhoA (Fig. 6B). No band was detected at the correct size for either Rac2 or RhoA.

As rhotekin is a strong binding partner of the GTPase, RhoA, a potential interaction with RTKN2 was tested. The stable RTKN2 cells were transiently transfected with constructs expressing constitutively active RhoA or Rac2 (a lymphocytic GTPase). Although all the proteins were successfully co-expressed neither RhoA nor Rac2 were co-precipitated with RTKN2 (Fig. 6).

### shRNA silencing of RTKN2 in CD4^+^ T cells

#### shRNA plasmid sequences and assessment of RTKN2 gene knockdown

The shRNA plasmids chosen for this study coded for four separate RTKN2 sequences within the full coding region of RTKN2 (Table 1). To measure the efficiency of the knockdown, a U1 promoter in the vector construct induced green fluorescent protein (GFP) expression in the cells. The RTKN2 shRNA plasmids were designated: shRNA nonsense sequence (NC), RTKN2 shRNA seq 1 (R1), RTKN2 shRNA seq 2 (R2), RTKN2 shRNA seq 3 (R3), RTKN2 shRNA seq 4 (R4), and the ‘no DNA’ control (NN). The efficiency of the gene knockdown by the shRNA plasmids was tested in human embryonic kidney (HEK 293T) cells that expressed low levels of endogenous RTKN2 mRNA. At 48 h post transfection the cells were processed for real time PCR. Although all four RTKN2 shRNA constructs induced GFP expression, the target gene expression was only silenced by R3 and R4 (50%–60% reduction, p < 0.05, data not shown). From these data two RTKN2 shRNA plasmids, R2 (no gene knockdown), and R4 (visible gene knockdown), were chosen for the nucleofection of CD4^+^ lymphocytes, as well as the NC and NN controls.

#### Nucleofection of CD4^+^ lymphocytes

**Figure 7:**
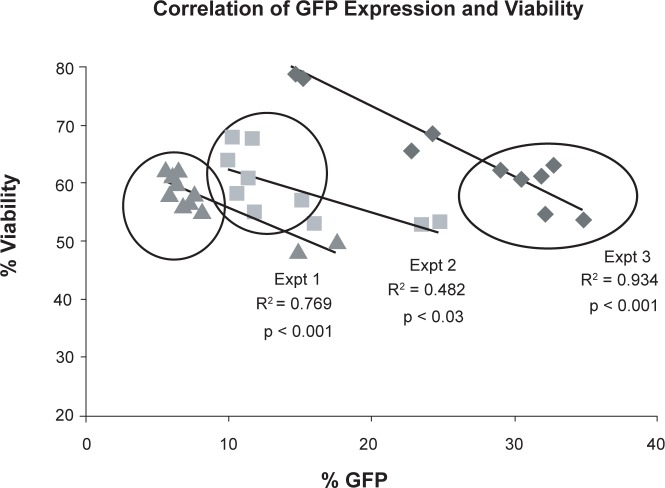
Inverse correlation of GFP expression in transfected CD4**^+^** cells with viability. GFP expression was estimated by flow cytometry in the cells transfected with shRNAs. The percentage of GFP positivity for all shRNA transfected CD4^+^ cells from three separate experiments were then plotted against cell viability. The r^2^ values indicated that as the GFP expression increases, the cell viability decreases. As viability was the end-point being studied this inverse correlation means that only cells with the same percentage GFP expression (within an experiment) were used to evaluate viability and the response to an oxysterol. The values selected are indicated by the drawn circles.

**Figure 8:**
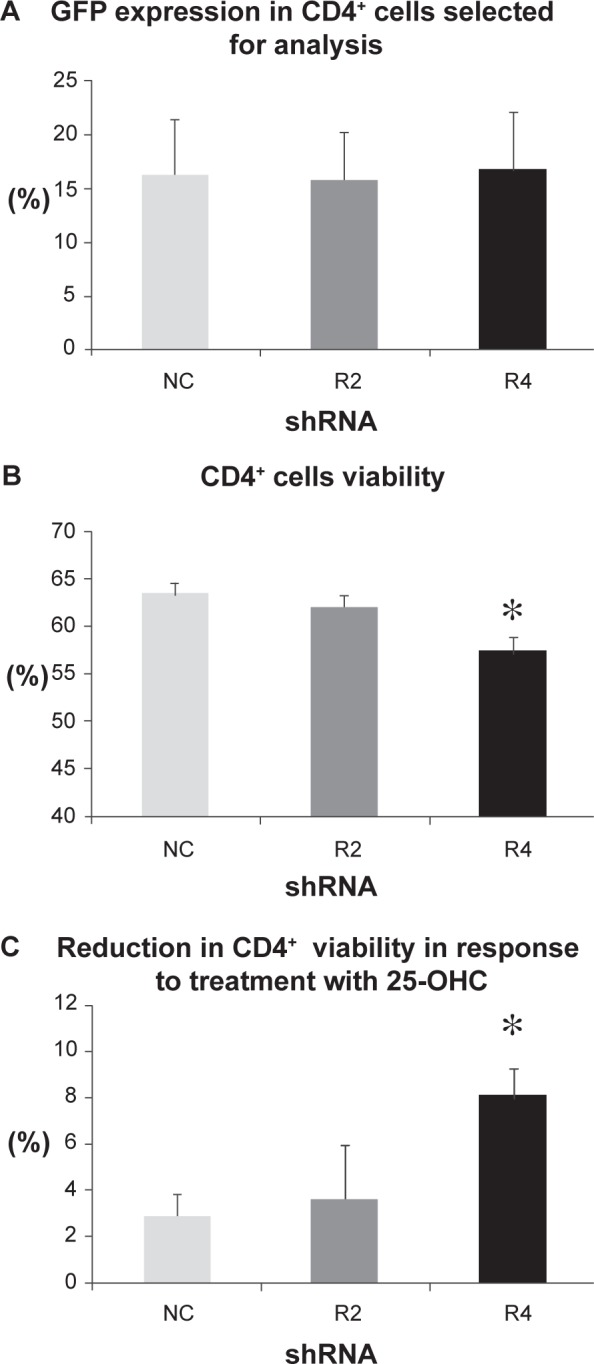
Transfected CD4**^+^** cells: GFP expression, cell viability and response to oxysterol. Results are expressed as mean ±SEM (n = 6 from 3 experiments). The GFP expression of the cells was measured using flow cytometry (FL-1 channel) 24 h following nucleofection. Cells that had been transfected with shRNAs and had similar GFP expression were selected for further analysis (Fig. 8A). Following 24 h culture post nucleofection, CD4^+^ T cells were removed and stained with 7-AAD. Using flow cytometry the percentage of 7-AAD-ve cells (FL-3 channel) was used to calculate the cell viability. The cells treated with the shRNA shown to silence RTKN2 (R4) had significantly lower viability than either the control shRNA (NC) or the non-silencing shRNA (R2) (p = 0.01, One-way ANOVA) (Fig. 8B). Following 24 h culture post nucleofection, CD4^+^ T cells were treated with/without 25-OHC for a further 24h before being removed and stained with 7-AAD. Using flow cytometry the percentage of 7-AAD-ve cells (FL-3 channel) was used to calculate the change in cell viability from untreated to OS treated. The cells treated with the shRNA shown to silence RTKN2 (R4) had a significantly greater drop in viability than the control shRNA (NC) (n = 3, p = 0.03, One-way ANOVA) (Fig. 8C).

CD4^+^ T-cells were isolated from umbilical cord blood and transfected either with NN or shRNA plasmids (NC, R2 and R4) using nucleofection technology. Twenty four hours following nucleofection, 7 AAD-ve viability and GFP expression were assessed by flow cytometry. It was noted in the first experiment that CD4^+^ T-cells transfected with the plasmid DNA had significantly lower viability (48.6% ±1.6% plasmid DNA (NC, R2 or R4) compared with 57.5% ±1.2% no DNA (NN), p < 0.05, n = 3). The GFP expression varied (from 6% to 35%) within experiments and between nucleofection pulses, and was found to inversely correlate with cell viability (Fig. 7). It was deduced that either DNA alone or the expression of GFP was having a marked effect on the cells. As viability was the end-point being studied, it was decided that when evaluating the effect of the shRNA it was important to select cells within each experiment with the same GFP expression—before comparing any other cellular changes. This meant that the average GFP expression for the cells selected for analysis was equal (NC, 16.2% ±5.1%; R2, 15.7% ±4.6%; R4, 16.8% ±5.4%, n = 6) (Fig. 8A).

#### Cell viability and response to oxysterol

After the cells were grouped and found to have statistically equivalent GFP expression, the viability of these subsets was assessed. Although RTKN2 was only silenced in a percentage of cells, the R4 group had significantly lower viability compared with both NC (control shRNA) and R2 (non-silencing shRNA) (p = 0.01, Fig. 8B). To test oxysterol sensitivity, cells that had been nucleofected and cultured for 24 h were then cultured in the presence/absence of 25-OHC, and the viability measured following 24 h treatment. At this time point (48 h post-nucleofection) 25-OHC reduced cell viability in all shRNA groups, but the reduction in cell viability was found to be significantly greater in R4 compared to NC and R2 (p = 0.03, Fig. 8C).

#### Gene analysis of silenced cells

**Figure 9:**
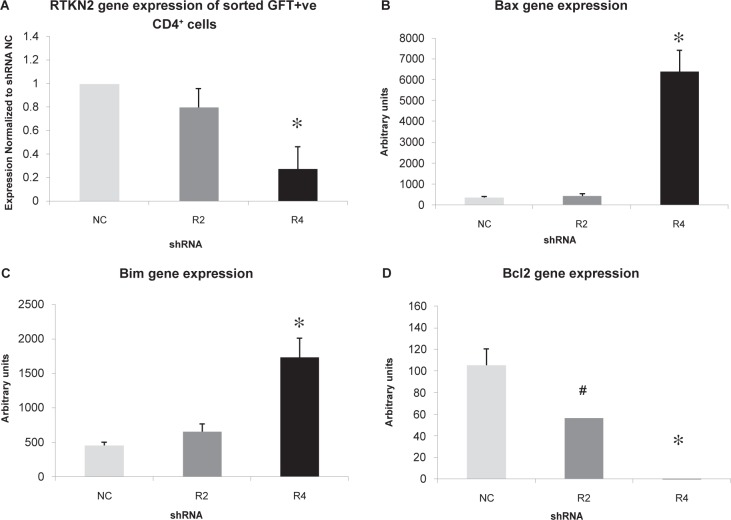
Gene analysis of the silenced CD4**^+^** cells. Results are expressed as mean ±SEM (n = 4). CD4^+^ cells were transfected with the shRNAs and cultured for 24 h before the viable cells were gated and fluorescent activated cell sorted (FACS) for GFP expression. To confirm gene silencing, real time PCR was performed with two different RTKN2 primer pairs and 2 different housekeeping genes, and normalized to the NC. The gene expression of RTKN2 was significantly silenced in shRNA (R4) (p = 0.044, One way ANOVA) but not in the scrambled control (NC) or the non-silencing control (R2) (Fig. 9A). To investigate expression of the BCL-2 family apoptotic genes, real time PCR was performed. The gene expression of pro-apoptotic genes, Bax and Bim, were significantly higher in R4 (*, p = 0.008 and 0.03 respectively, One way ANOVA) (Figs. 9B and 9C) compared to NC and R2, while Bcl2 expression was significantly reduced (*, p = 0.011, One way ANOVA) (Fig. 9D). The Bcl2 expression in R2 appeared reduced compared to the NC control but it was not statistically significant (#, p = 0.08, One way ANOVA, Tukey’s Pairwise Comparison) (Fig. 9D).

To verify gene knockdown in the transfected CD4^+^ T-cells, viable GFP positive cells were fluorescent activated cell sorted (FACS) using a BD FACSAria™ cell sorter and processed for real time PCR. When the values were normalized to NC, the expression of RTKN2 in R4 was significantly lower than both NC and the non-silencing R2 (p < 0.05, Fig. 9A). As the sample size was small it was not possible to measure protein levels. However in order to determine possible involvement of genes regulating apoptotic signaling pathways, the expression levels of Bcl2 family members, Bax, Bim (pro-apoptotic) and Bcl2 (anti-apoptotic) were examined in the silenced CD4^+^ sorted samples. There was a significant increase in the gene expression of both Bax (p = 0.008, Fig. 9B) and Bim (p = 0.03, Fig. 9C) genes in the RTKN2 silenced cells, while in contrast, Bcl2 expression was significantly reduced in the silenced RTKN2 cells (R4) (p = 0.011, Fig. 8D). It was noted that Bcl2 expression was also slightly lower in the non-silencing group (R2) (Fig. 9D). Although this did not reach significance, it may have been due to a very low level of RTKN2 knockdown (Fig. 9A).

## Discussion

The original identification of RTKN2 in a cell model that had been induced to become resistant to the oxysterol, 25-OHC^1^,^12^ indicated an association with the process of apoptosis. In the study described here we have manipulated gene and protein levels and demonstrated a direct role of RTKN2 in cell survival and apoptosis. The response of the stable RTKN2 over expressing HEK cells to three apoptotic agents differed according to the mode of action of the inhibitory compound, and thereby suggested the potential pathways by which RTKN2 may act. RTKN2 cells were resistant to apoptotic agents that acted via the intrinsic mitochondrial pathways (oxysterol, 25-OHC, and camptothecin) but sensitive to the extrinsic agent, TNFα. 25-OHC is known to induce apoptosis through down-regulation of BCL-2 expression and activation of caspases,^17^ while camptothecin is a topo-isomerase inhibitor that interferes with transcription, induces DNA strand breaks and inhibits DNA division.^18^ The nuclear stress caused by camptothecin then activates the intrinsic mitochondrial apoptotic pathway.^19^

The resistance to 25-OHC and camptothecin by the RTKN2 cells indicated that RTKN2 expression favours an environment in which the cell is able to withstand mitochondrial death signals. The combination of pathway inhibitors and 25-OHC then showed that the RTKN2 oxysterol resistance was NF-KappaB pathway dependent. This role of NF-KappaB was then confirmed by the increased steady-state activation of the pathway in the RTKN2 cells. NF-KappaB is responsible for transcription or repression of many genes including the mitochondrial death genes^20^,^21^ and down regulation of pro-apoptotic genes, Bax and Bim, in the HEK RTKN2 cells is consistent with this action and pro-survival. Although there was no significant change in BCL-2 expression between the two cell lines, as Bax inhibits the initial step of mitochondrial outer membrane permeabilization^22^ a lower expression would be adequate for inhibition of cell death.

In contrast to the intrinsic agents, RTKN2 was sensitive to the extrinsic apoptotic action of TNFα. Cell death from TNFα occurs after engagement of the death receptor with induction of Fas-associated death domain-dependent (FADD) caspase-8 activation. In addition TNFα activates the NF-kappaB pathway that is known to promote cell survival.^23^–^25^ The resultant TNFα signaling is complex, and in instances of apoptosis, the caspase pathway outweighs the NF-kappaB signaling pathway.^26^,^27^ Although the pathway inhibitor experiment showed that the resistance seen in the RTKN2 cells was NF-kappaB dependent, the mediated survival effects appeared not to be at a level that could counteract the apoptotic induction of TNFα at 48 h. However, consistent with NF-KappaB activation, it was observed that RTKN2 cells were significantly resistant to TNFα at the earlier 24 hour time point (data not shown) but this difference had disappeared by 48 h.

The NF-KappaB dependent inhibition of apoptosis was also seen in the other family member, rhotekin over expressing cells. When cell death was induced by serum deprivation or treatment with sodium butyrate, the rhotekin-mediated anti-apoptotic effect appeared to act via RhoA and, like RTKN2, was blocked by NF-kappaB inhibitors.^10^ As rhotekin is known to bind to RhoA with very high affinity^28^ we used the HEK stable cells to determine whether RTKN2 also interacted with the same GTPase. Rac2 was chosen as another potential binding partner due to its high expression in lymphocytes.^29^,^30^ In the experiments performed in this study, neither RhoA nor Rac2 bound to RTKN2 in levels that were detectable in the co-immunoprecipitated proteins. However as RTKN2 has only 47% identical amino acid homology to rhotekin in their HR-1 (Rho-binding) domains and 65% in the overall protein, RTKN2 may not interact with the same proteins as rhotekin. Indeed, a recent paper noted that proteins can have 88% identity but still exhibit contrasting functions and structure,^31^ and it is possible that RTKN2 binds with more affinity to one of the other Rho-GTPases or GTPase family members. Other potential candidates include members of the GTPase Immune Associated Protein (GIMAP) family, GTPases that have been recently identified and play a specific role in survival, cell cycle and apoptosis in lymphocytes from the human, mouse and rat.^32^

Having shown that over-expression of RTKN2 conferred resistance to apoptosis we then investigated its role in the endogenously expressing human CD4^+^ T-cells using gene knockdown. Nucleofection, an electroporation-based method, was used as it has previously been shown to be useful in cells that are difficult to transfect, including hematopoietic cells such as NK cells,^33^ primary mouse CD4^+^ cells^34^ and human CD4^+^ T cell cultures.^35^ As expected, gene knockdown of RTKN2 had the opposite effect to over expression of the protein. Although only a percentage of the cells were silenced there was a small but significant decrease in the cell viability of the RTKN2 silenced CD4^+^ T-cells; and furthermore, this population of cells was more sensitive to the apoptotic effect of the oxysterol, 25-OHC. The gene expression of the anti-apoptotic member, BCL-2,^36^,^37^ and pro-apoptotic members, Bax and Bim was measured in the RTKN2 silenced T-cells and their regulation correlated to the behaviour of the cells and the expected phenotype for dying cells. The ratio of anti- and pro-apoptotic BCL-2 family members has been shown specifically to regulate T-cells from apoptotic-resistant towards an apoptosis-sensitive state.^38^ As BCL-2 not only reduces apoptosis but has also been shown to retard cell cycle entry^39^ RTKN2 role in lymphocyte quiescence will be investigated in the future. The mitochondrial regulated pathway is essential for spontaneous T-cell death^32^,^40^ and as RTKN2 was able to protect both HEK cells and CD4^+^ T-cells from this type of apoptosis this indicates it may play an important role in the life cycle of a T-lymphocyte.

RTKN2 belongs to a large family of effector proteins with GTPase binding domains.^41^ They are critical for the downstream signaling that follows binding to an activated GTPase^42^ and in some instances are cell specific.^41^,^43^ Many of the GTPase proteins and their effectors have previously been linked to both suppression and promotion of apoptosis^44^ dependent on the balance of multiple factors including; cell type, GTPase, activating proteins (GAPs), and the specific effectors.^41^ The studies presented here demonstrate that the over expression of the full length RTKN2 resulted in gene changes that protected HEK cells or induced sensitivity of CD4^+^ T-cells to selected mitochondrial apoptotic stimuli. In the HEK cells the resistance to apoptosis was dependent on NF-KappaB signaling and in CD4^+^ cells RTKN2 was associated with direct regulation of genes that correlated with a pro-death phenotype. In conclusion we have shown that RTKN2 plays a similar anti-apoptotic role to rhotekin but not through interaction with RhoA. The pathway by which RTKN2 protects the cell is NF-KappaB dependent and involves regulation of the mitochondrial genes, Bax, Bim and BCL-2 in the T-cells. Being that the gene is specifically expressed in CD4^+^ cells we hypothesize that RTKN2 is a lymphocyte GTPase effector that plays a role in signal control of T-cell survival/fate by regulation of pro-apoptotic and anti-apoptotic BCL-2 genes.

## Methods

### Ethics approval

Umbilical cord blood samples were donated in accordance with approved institutional guidelines, and with appropriate ethics approval.

### Transfection of mammalian cells

To establish a stable cell line, HEK Flip-In™-293 (Human embryonic kidney cells (Invitrogen) containing a single stably integrated FRT (Flp Recombinase Target site, Invitrogen) were utilized. A pcDNA5/FRT construct either containing the gene of interest (pcDNA5/FRT-His-V5-RTKN2) or control gene (pcDNA5/FRT/V5-His/CAT) were co-transfected with pOG44™ expression plasmid (allowing expression of Flp recombinase and integration of the pcDNA5/FRT plasmid within the genome, Invitrogen) into the host-cell line (Flip-In™-293). Incorporation of the pcDNA5/FRT construct into the genome occurs at the FRT site in the Flip-In™-293 and the pcDNA5/FRT plasmid contains the Hygromycin resistance gene which allows the selection of stable cell lines. The control construct (as recommended by the manufacturer) expressed the hygromycin resistant gene and the recombinant protein chloramphenicol acetyltransferase (CAT) and has previously been used as a valid control.^45^ Transfection was carried out according to standard protocol and media containing 600 μg/ml of hygromycin was used to select for CAT or RTKN2 expressing cells. Single colonies were then removed using a fine tip pipette and a dissecting microscope, and re-seeded in separate wells of a 24 well plate. Confluent cells were then expanded and maintained in frequent changes of hygromycin (Invitrogen, 600 μg/ml) standard HEK media (high glucose (4.5 mg/L D-glucose) DMEM (Invitrogen) with Glutamax™ and 10% FCS). Colonies were selected and established; with 2 control cell lines, CAT#1 and CAT#2, designated CAT control cells, and two with the gene of interest, RTKN2#1 and RTKN2#2, designated RTKN2 cells.

### Western blot analysis

CAT control and RTKN2 cells were lysed in MPER (Pierce) for 10 min and centrifuged at 12000 × g for 10 min at 4 °C. The clarified supernatant was separated from the pellet and stored for protein measurement and analysis. A total of 20 ug of total protein was loaded in each lane of the 4%–20% pre-cast Tris Glycine PAGE gels (GE Healthcare) and transferred on to PVDF membrane (iBlot Dry Blotting System, Invitrogen). The tagged RTKN2 full length protein (comprising both the GTPase-binding and PH domains) and the control CAT protein were visualized by western blot analysis using mouse anti-V5 (1:5000) (Invitrogen), and HRP-conjugated anti-mouse IgG (1:2000) (Cell Signaling) and then visualised using ECL plus detection reagents (Amersham) as per manufacturer’s instructions and imaged using the UVP ChemiDoc-It Imaging System (DKSH). Other antibodies used to determine NF-KappaB activation were anti-phospho NF-KappaB p65 (Cell Signaling Technology, #3033) and anti beta-actin (Abcam, ab6276).

### Induction of apoptosis

For apoptotic treatments, stock solutions of 25-hydroxy cholesterol (25-OHC), tumour necrosis factor alpha (TNFα), and camptothecin (all purchased from Sigma) were freshly prepared in DMSO (TNFα and camptothecin) and ethanol (25-OHC). Working solutions were made up in media in which the doses varied depending on the treatment. Apoptosis was induced in cells using a range of doses; 2.5 μg/ml, 5 μg/ml, 10 μg/ml of 25-OHC, 10 μM, 20 μM, and 30 μM of camptothecin and 10 ng/ml, 20 ng/ml, and 30 ng/ml of TNFα, with all treatments having equal amounts of the DMSO or ethanol carrier. Following trypsinization, the duplicate cell lines for CAT control and RTKN2 cells were cultured in triplicate in flow tubes that were cultured on rollers at 37 °C. This was done to allow the effective measure of apoptotic cells without the interference of trypsin on the cell membrane. At 48 h samples were removed for total RNA extraction, and the remaining cells were used to assess the viability and level of apoptosis using Annexin V-FITC (Becton Dickson) and propidium iodide (PI, Molecular Probes).

### Apoptosis and pathway inhibitors

For inhibitor treatments, stock solutions of curcumin, parthenolide and PD98059 were freshly prepared in DMSO. Working solutions were made up in media to a known inhibitory dose, with curcumin 10 uM, parthenolide 10 uM and PD98059 7.5 uM. Apoptosis was induced as described above using 5 ug/ml 25-OHC in combination with the various inhibitors and at 48 h aliquots of cells were removed to assess the viability and level of apoptosis using Annexin V-FITC and PI.

### Measurement of apoptosis

Annexin V binds to the phosphatidyl serine of the cell membrane in cells undergoing apoptosis, and propidium iodide (PI) intercalates with the DNA so that in combination the dyes can discriminate dead and dying cells. In brief approximately 4 × 10^5^ cells were diluted with PBS to wash and centrifuged at 2500 rpm. Cells were then washed in 1 × Annexin V Binding Buffer (10 mM Hepes/NaOH, pH 7.4, 140 mM NaCl, 2.5 mM CaCl_2_) and stained using Annexin V FITC (5 μM per tube) and PI (10 μM) for 15 minutes in the dark. Before flow cytometry analysis (CELLQuest software) 400 μl of binding buffer was added to each sample. The percentage of apoptotic and dying cells (Annexin V FITC +ve and PI +ve) was determined by region analysis.

### Total RNA isolation, DNase treatment and reverse transcription

For gene expression studies total RNA was extracted using Trizol^®^ (Invitrogen) reagent, and the quantity of the RNA was determined by UV spectrophotometry. Contaminating genomic DNA was removed by digestion with RQ1 RNase-Free DNase (Promega) (30 minutes, 37 °C before inactivation of enzyme at 99 °C), and cDNA was prepared by reverse transcription using random primers and the Reverse Transcription System (Promega) according to the manufacturers guidelines.

### Real time assays on demand

A number of pre-designed Real-Time TaqMan^®^ Gene Expression Assay mixes (Applied Biosystems) were purchased for semi-quantitative PCR. These included RTKN2, (Hs00377175), Bax (Hs00751844), Bim (Hs00708019), and BCL-2 (Hs00608023), and the expression was compared to both housekeeping genes, Beta-Actin and GAPDH.

### Transient transfection and immunoprecipitation

The constructs, pcDNA3 RhoA (a gift from Dr. Wei Duan, Deakin University), and pcDNA3 Rac2 (UMR cDNA Resource Centre), expressing constitutively active forms of the respective GTPase proteins were transiently transfected into the stable RTKN2 HEK cells. To perform the transfection, cells were split to a density that reached 80%–90% confluence after 48 hours culture. Cells were transfected using Lipofectamine LTX (Invitrogen) and after 48 h an aliquot of each cell lysate (pre-IP) was removed and stored (−80 °C) for confirmation of expression of the recombinant proteins. The remaining samples were pre-cleared using either anti-mouse or rabbit immunoglobulin (IgG). Immunoprecipitation was then immediately performed with mouse-anti-V5 (RTKN2 pull-down) antibodies and precipitated using A/G Sepharose beads (Santa Cruz Biotechnology). The co-immunoprecipitated proteins were washed a number of times with lysis buffer and after addition of reducing buffer they were then heated at 99 °C to separate the proteins and stored at −80 °C until western blot analysis. The western blot was performed as described above using the antibodies, mouse-anti-V5 (RTKN2) (Invitrogen), rabbit-anti-RhoA (Cell Signaling) or rabbit-anti-HA (Rac2) (Zymed Laboratories).

### Preparation of hematopoietic mononuclear cells and immuno-magnetic separation of CD4^+^ T-cells

Mononuclear cells (MNC) from umbilical cord blood were prepared by density gradient separation using Ficoll-Paque™ PLUS (GE Healthcare Amersham Biosciences, Uppsala, Sweden) according to the manufacturer’s instructions. In brief, the samples were diluted with an equal volume of phosphate buffered saline, pH 7.4 (PBS), layered on to the Ficoll-Paque™ PLUS and centrifuged for 25 minutes at 440 g. Following centrifugation, the MNC, which were concentrated in a ‘fuzzy’ layer at the Ficoll-Paque™ PLUS-plasma interface, were collected into sterile tubes and washed twice in at least double their volume of PBS. To obtain lymphocytes, the MNC’s were allowed to adhere to the flask (3 × 10^7^ MNC per 25 cm^2^ flask) for 2 h in RPMI with 15% FCS and the non-adherent cells removed for CD4^+^ isolation.

For separation of the T-cell subset, the non-adherent cells were labelled with CD4-microbeads and positively selected using immuno-magnetic labelling and MACS^®^ Technology (Miltenyi Biotec GmbH, Germany) according to the manufacturer’s instructions. The viability of the cells was assessed using Trypan Blue staining and purity of the population determined by flow cytometric analysis.

### shRNA plasmids

Five pre-designed short hairpin (sh) RNA plasmids were supplied by Superarray SureSilencing™ to specifically knock down the expression of RTKN2 by RNA interference. Four of the shRNA plasmids (R1–R4) comprised different regions of the RTKN2 gene sequence, while the fifth shRNA was a negative control (NC) consisting of a scrambled artificial sequence with no sequence identity to the human genome. Each plasmid was incorporated with the pGeneClip™ hMGFP gene controlled by the U1 promoter, for identification and potential enrichment of the transiently transfected cells. The expression of GFP aided in estimating transfection efficiencies by flow cytometry. SuperArray SureSilencing™ guaranteed that at least two of the four RTKN2 shRNA’s would knock down at least 70% of the targeted gene (following 100% transfection efficiency).^46^ The supplied shRNA plasmids were received freeze-dried and reconstituted in 200 μl of Tris EDTA (Sigma), (pH 8.0) buffer. Chemically competent cells were transformed with the plasmid and high quality midi-preparations of plasmid DNA were then prepared.

### Efficiency of RTKN2 knockdown

Equal concentrations of the shRNA plasmids were prepared for transfection so that the diluted DNA introduced into the cells was of equal volume. To test the efficiency of the plasmids, standard HEK 293T cells were transfected, cultured for 48 h (the time recommended by the manufacturer for visible gene knockdown) and GFP expression measured by flow cytometry. The remaining cells were lysed with Trizol, total RNA extracted and cDNA synthesised. The RTKN2 gene expression was then determined by real time PCR.

### Nucleofection of CD4**^+^** T-cells

Nucleofection technology (AMAXA Biosystems^©^) is a recent improved form of electroporation that utilises a proprietary piece of equipment, the Nucleofector. Specific kits for each cell type have been developed and allow even non-dividing primary cells such as resting T lymphocytes to be transfected. The karyophilic solution provided forms a complex with the plasmid DNA allowing transport across the nuclear envelope via the nuclear pores. The CD4^+^ T-cells were isolated and nucleofection was carried out according to manufacturers instructions. In summary, approximately 5–10 × 10^6^ CD4^+^ T-cells were mixed in a certified cuvette with 100 μl/sample of the appropriate cell-type specific Nucleofector Solution and 3 μg of shRNA plasmid. The appropriate Nuclefector program was set for high viability (V-24/V-O24) and after the electric pulse, cells were re-suspended in 500 μl of RPMI/FCS media. Cells were then transferred to a culture dish with an extra 1.5 ml of RPMI/FCS media and incubated for 6 h at 37 °C, after which the cells were washed and re-suspended in 2 ml of RPMI/10% FCS. Cell viability and GFP expression were assessed at 24 h and 48 h using flow cytometry. In some experiments the cells were divided into two groups after 24 h and treated with the oxysterol, 25-OHC or ethanol alone and cultured a further 24 h.

### 7-AAD and GFP expression

Cells were stained with 7-AAD (a fluorescent vital dye that binds DNA and does not cross intact plasma membranes) to enable identification of the viable and non-viable cells. Cells were assessed on a FACSCalibur flow cytometer (Becton Dickinson) and to determine GFP expression in viable cells, 7-AAD-ve stained cells were gated and the percentage of cells that had shifted into the FL-1 (GFP+ve) channel compared to the negative controls. Analysis was performed using the CellQuest Pro software.

### Sorting of GFP+ve viable CD4^+^ T-cells

The BD FACSAria™ (Becton Dickinson) cell sorter was utilized to gate the viable CD4+ T-cells following 24 h culture and specifically sort GFP+ve cells from GFP–ve cells. The sorted cells were re-suspended in PBS and then spun down, the supernatant aspirated and the cells lysed in Trizol for RNA extraction and cDNA transcription and real time PCR for semi-quantitative expression of RTKN2.

### Statistics

Data was expressed as mean ±SEM. To analyse statistical differences the assumption of normality was made for the data, and the General Linear Model (GLM) performed to compare between treatments and treatment groups. A Tukey’s multiple comparison testing was performed for pairwise comparisons.

## Figures and Tables

**Table 1:** Superarray shRNA RTKN2 sequences.

shRNA	shRNA insert sequence (5′–3′)
R1	GCCAGGAGAAATAGATTAAGT
R2	CCTGCAAGTGAGCCATTACAT
R3	AGAGGAAATTGAAGCTAAAGT
R4	GCAACCTCAGTCTACCATGAT
NC	GGAATCTCATTCGATGCATAC
